# Supplementary material for: Disentangling the effects of traits with shared clustered genetic predictors using multivariable Mendelian randomization
Source: Genet Epidemiol. 2022 May 31;46(7):415–29. doi: 10.1002/gepi.22462 (PMC9541575; doi:10.1002/gepi.22462)
Supplement: Supplementary file 1 — Supplementary Information [file GEPI-46-415-s001.pdf]

Supplementary Material

A.1 Software code for MV-IVW and MV-IVW-PCA methods

We provide R code to implement the MV-IVW and MV-IVW-PCA methods with three exposure traits. Genetic associations with the exposure are denoted `beta_x1`, `beta_x2`, and `beta_x3`, and their standard errors `se_x1`, `se_x2`, and `se_x3`. Genetic associations with the outcome are denoted `beta_y` and their standard errors `se_y`. The variant correlation matrix is denoted `rho`. Code is supplied both using the *MendelianRandomization* package, and using matrix calculations directly:

```
# MV-IVW method
###############
# note use of fixed-effect model under assumption that all variants in same
# genetic region and hence influence the outcome through same causal pathway

library(MendelianRandomization)
mvivw = mr_mvivw(mr_mvinput(cbind(beta_x1, beta_x2, beta_x3),
  cbind(se_x1, se_x2, se_x3), beta_y, se_y, correl=rho), model="fixed")
mvivw$Estimate; mvivw$StdError

beta_x    = cbind(beta_x1, beta_x2, beta_x3)
Sigma     = se_y%o%se_y*rho
mvivw_est = solve(t(beta_x)%*%solve(Sigma)%*%beta_x)%*%t(beta_x)%*%solve(Sigma)%*%beta_y
mvivw_se  = sqrt(diag(solve(t(beta_x)%*%solve(Sigma)%*%beta_x)))

# MV-PCA method
###############

Psi = ((abs(beta_x1)+abs(beta_x2)+abs(beta_x3))/se_y)%o%
      ((abs(beta_x1)+abs(beta_x2)+abs(beta_x3))/se_y)*rho
K   = which(cumsum(prcomp(Psi, scale=FALSE)$sdev^2/
              sum((prcomp(Psi, scale=FALSE)$sdev^2)))>0.99)[1]
       # K is number of principal components to include in analysis
       # this code includes principal components to explain 99% of variance in the risk factor
betaXG1   = as.numeric(beta_x1%*%prcomp(Psi, scale=FALSE)$rotation[,1:K])
betaXG2   = as.numeric(beta_x2%*%prcomp(Psi, scale=FALSE)$rotation[,1:K])
betaXG3   = as.numeric(beta_x3%*%prcomp(Psi, scale=FALSE)$rotation[,1:K])
betaYG0   = as.numeric(beta_y%*%prcomp(Psi, scale=FALSE)$rotation[,1:K])
sebetaYG0 = as.numeric(se_y%*%prcomp(Psi, scale=FALSE)$rotation[,1:K])
Sigma     = se_y%o%se_y*rho
pcSigma   = t(prcomp(Psi, scale=FALSE)$rotation[,1:K])%*%Sigma%*%
              prcomp(Psi, scale=FALSE)$rotation[,1:K]

mvpca     = mr_mvivw(mr_mvinput(cbind(betaXG1, betaXG2, betaXG3),
  cbind(rep(1, length(betaXG1)), rep(1, length(betaXG1)), rep(1, length(betaXG1))),
                   betaYG0, rep(1, length(betaXG1)), corr=pcSigma), model="fixed")
mvpca$Estimate; mvpca$StdError
# note that the standard errors of the genetic associations with the exposures are
# not used in the calculation, and so are set to 1

mvpca_est = solve(rbind(betaXG1, betaXG2, betaXG3)%*%solve(pcSigma)%*%
                  cbind(betaXG1, betaXG2, betaXG3))%*%
                  rbind(betaXG1, betaXG2, betaXG3)%*%solve(pcSigma)%*%betaYG0
mvpca_se  = sqrt(diag(solve(rbind(betaXG1, betaXG2, betaXG3)%*%solve(pcSigma)%*%
                        cbind(betaXG1, betaXG2, betaXG3))))
```

A.2 Software code for MV-LIML and MV-LIML-PCA methods

We provide R code to implement the MV-LIML and MV-LIML-PCA methods. The matrix of genetic associations with the exposure is denoted `Bx`, and standard errors `Sx`. The vector of genetic associations with the outcome is denoted `By` and standard errors `Sy`. The variant correlation matrix is denoted `rho`, the trait correlation matrix is denoted `Phi`, and the number of principal components R; this can be provided by the user, or estimated automatically:

```
# MV-LIML method
#################

mvmr_liml <- function(Bx,By,Sx,Sy,rho,Phi) {
Bx <- as.matrix(Bx);   By <- as.vector(By)
Sx <- as.matrix(Sx);   Sy <- as.vector(Sy)
rho <- as.matrix(rho); Phi <- as.matrix(Phi)
J <- ncol(Bx); K <- nrow(Bx)

GamY <- (Sy%*%t(Sy))*rho

gamX <- list()
for (k in 1:J){
  gamX[[k]] <- (Sx[,k]%*%t(Sx[,k]))
}

GamX1 <- function(tet){
gamX.t  <- list()
for (k in 1:J){
  for (l in 1:J){
    gamX.t[[(((k-1)*J)+l)]] <- sqrt(gamX[[k]])*sqrt(gamX[[l]])*rho*tet[k]*tet[l]*Phi[k,l]
  }
}
return(gamX.t)
}

GamX <- function(tet){Reduce('+',GamX1(tet))}

# LIML estimation
g <- function(tet){as.vector(By - (Bx%*%tet))}
Om <- function(tet){as.matrix(GamY + GamX(tet))}
Q <- function(tet){as.numeric(t(g(tet))%*%(solve(Om(tet)))%*%g(tet))}
G <- -as.matrix(Bx)
DQ <- function(tet){2*as.matrix(t(G)%*%(solve(Om(tet)))%*%g(tet))}
liml <- nlminb(rep(0,J),objective=Q,gradient=DQ)
est <- as.vector(liml$par)
var.est <- as.matrix(solve(t(G)%*%(solve(Om(est)))%*%G))

res.list <- list("est"=est, "var"=var.est)
return(res.list)
}
```

```
# MV-PCA-LIML method
####################

mv_pca_liml <- function(Bx,By,Sx,Sy,rho,Phi,R=NULL) {
Bx  <- as.matrix(Bx);   By <- as.vector(By)
Sx  <- as.matrix(Sx);   Sy <- as.vector(Sy)
rho <- as.matrix(rho); Phi <- as.matrix(Phi)
J <- ncol(Bx); K <- nrow(Bx)
Psi = (rowSums(abs(Bx))/Sy)%o%(rowSums(abs(Bx))/Sy)*rho

# estimate number of principal components
if(missing(R)) {
  R=which(cumsum(prcomp(Psi,scale=FALSE)$sdev^2/sum((prcomp(Psi,scale=FALSE)$sdev^2)))>0.99)[1]
} else {  R=R  }
lambda <- sqrt(K)*(eigen(Psi)$vectors[,1:R])
evec <- eigen((t(lambda)%*%lambda))$vectors
eval <- eigen((t(lambda)%*%lambda))$values
lambda <- lambda%*%(solve(evec%*%diag(sqrt(eval))%*%t(evec)))
dim(lambda) <- c(K,R)

GamY <- (Sy%*%t(Sy))*rho

gamX <- list()
for (k in 1:J){
  gamX[[k]] <- (Sx[,k]%*%t(Sx[,k]))
}

GamX1 <- function(tet){
gamX.t  <- list()
for (k in 1:J){
  for (l in 1:J){
    gamX.t[[(((k-1)*J)+l)]] <- sqrt(gamX[[k]])*sqrt(gamX[[l]])*rho*tet[k]*tet[l]*Phi[k,l]
  }
}
return(gamX.t)
}

GamX <- function(tet){Reduce('+',GamX1(tet))}

# LIML estimation
g <- function(tet){as.vector(t(lambda)%*%(By - (Bx%*%tet)))}
Om <- function(tet){as.matrix(t(lambda)%*%(GamY + GamX(tet))%*%lambda)}
Q <- function(tet){as.numeric(t(g(tet))%*%(solve(Om(tet)))%*%g(tet))}
G <- -as.matrix(t(lambda)%*%Bx)
DQ <- function(tet){2*as.matrix(t(G)%*%(solve(Om(tet)))%*%g(tet))}
liml <- nlminb(rep(0,J),objective=Q,gradient=DQ)
est <- as.vector(liml$par)
var.est <- as.matrix(solve(t(G)%*%(solve(Om(est)))%*%G))

res.list <- list("est"=est, "var"=var.est)
return(res.list)
}
```

A.3 Additional simulation results

In addition to the main simulation study, we also consider the performance of the MV-IVW-PCA and MV-LIML-PCA methods with other parameter settings:

- Supplementary Table A1. Weaker instruments: we generate the α_j parameters from a normal distribution with mean 0.5.

- Supplementary Table A2. Stronger correlations: we generate elements of the A matrix from a uniform distribution on +0.1 to +1.0. Note that as the vast majority of correlations between variants were above +0.6, we did not attempt the methods that rely on pruning in this scenario.

- Supplementary Table A3. Stronger effects: we set $\theta_1 = +0.8$ and $\theta_3 = +1.0$.

- Supplementary Table A4. Alternative approach for generating a correlation matrix 1: the "c-vine" method from the R package *clusterGeneration*. The code is:
  ```
  sig <- cor(genPositiveDefMat(dim=vars, covMethod = "c-vine",
     eigenvalue=runif(vars, -1, 1))$Sigma).
  ```
  Correlations typically ranged from around −0.5 to +0.5 with an interquartile range from around −0.1 to +0.1.

- Supplementary Table A5. Alternative approach for generating a correlation matrix 2: the "onion" method from the R package *clusterGeneration*. The code is:
  ```
  sig <- cor(genPositiveDefMat(dim=vars, covMethod = "onion",
     eigenvalue=runif(vars, -1, 1))$Sigma)
  ```
  Correlations typically ranged from around −0.5 to +0.5 with an interquartile range from around −0.1 to +0.1.

- Supplementary Table A6. Correlations were obtained from data on participants of European ancestries from UK Biobank. After pruning at a threshold of $r^2 < 0.95$, we took correlations from the first 100 variants in the chemokine receptor gene cluster considered in the applied analysis.

- Supplementary Table A7. Exposure traits were correlated by adding an additional term U_{i2} to the equation for the generation of X_{i1} to read $X_{i1} = \sum_{j=1}^{5} \alpha_j G_{ij} + U_{i1} + U_{i2} + \epsilon_{Xi1}$. Similarly, we added U_{i3} to X_2, and U_{i1} to X_3. This correlation was not accounted for in the MV-LIML method.

- Supplementary Table A8. Exposure traits were correlated as in the previous scenario, except that the equation for the generation of X_{i1} read $X_{i1} = \sum_{j=1}^{5} \alpha_j G_{ij} + U_{i1}/\sqrt{2} + U_{i2}/\sqrt{2} + \epsilon_{Xi1} \times \sqrt{2}$, and similarly for X_2 and X_3. This ensures that the variances of X_1, X_2, and X_3 remained the same as in the previous scenario, but correlations between the exposures were weaker.

- Supplementary Table A9. Exposure traits were correlated as in the previous two scenarios, except that the equation for the generation of X_{i1} read $X_{i1} = \sum_{j=1}^{5} \alpha_j G_{ij} + U_{i1} \times \sqrt{1.4} + U_{i2} \times \sqrt{1.4} + \epsilon_{Xi1} \times \sqrt{0.2}$, and similarly for X_2 and X_3. This ensures that the variances of X_1,X_2, and X_3 remained the same as in the previous two scenarios, but correlations between the exposures were stronger.

- Supplementary Table A10. Smaller independent sample for variant correlation matrix: the correlation matrix between genetic variants is estimates based on an independent sample of 1000 individuals.

In each setting, the proposed PCA methods performed well, with close to nominal Type 1 error rates in most scenarios and greater power than for the MV-IVW and MV-LIML methods at pruning thresholds where those methods maintained reasonable Type 1 error rates.

Supplementary Table A1: Results from simulation study with weaker instruments

Parameter	Method	Pruning	Mean	SD	Mean SE	Power
θ_1	MV-IVW	Oracle	0.297	0.196	0.180	39.2
		0.4	0.215	0.225	0.204	22.3
		0.6	0.119	0.132	0.119	20.5
		0.8	−0.050	0.505	0.066	71.9
	MV-LIML	Oracle	0.349	0.237	0.201	43.0
		0.4	0.276	0.312	0.229	28.8
		0.6	0.215	0.266	0.128	42.5
		0.8	0.106	2.425	0.234	75.2
	MV-IVW-PCA	-	0.208	0.176	0.164	26.5
	MV-LIML-PCA	-	0.296	0.262	0.182	41.2
θ_2	MV-IVW	Oracle	−0.012	0.197	0.180	6.9
		0.4	−0.022	0.228	0.205	7.0
		0.6	−0.012	0.134	0.119	8.0
		0.8	−0.043	0.499	0.065	72.0
	MV-LIML	Oracle	−0.006	0.238	0.201	7.5
		0.4	−0.015	0.315	0.230	9.9
		0.6	−0.002	0.274	0.128	22.4
		0.8	0.015	2.437	0.234	75.4
	MV-IVW-PCA	-	−0.023	0.176	0.164	6.7
	MV-LIML-PCA	-	−0.014	0.264	0.182	13.9
θ_3	MV-IVW	Oracle	−0.478	0.196	0.180	75.4
		0.4	−0.381	0.233	0.206	50.3
		0.6	−0.204	0.148	0.119	44.3
		0.8	−0.181	0.500	0.066	72.8
	MV-LIML	Oracle	−0.539	0.233	0.202	78.2
		0.4	−0.455	0.317	0.231	56.5
		0.6	−0.326	0.275	0.128	64.4
		0.8	−0.012	2.283	0.233	74.7
	MV-IVW-PCA	-	−0.368	0.179	0.165	61.3
	MV-LIML-PCA	-	−0.475	0.263	0.182	72.3

Mean estimates, standard deviation (SD) of estimates, mean standard error (mean SE) of estimates, and empirical power of the 95% confidence interval to estimate $\theta_1 = 0.4$, $\theta_2 = 0$, and $\theta_3 = -0.6$. We consider four methods, and various pruning thresholds for the MV-IVW and MV-LIML methods, plus an oracle setting in which only the 15 variants that truly affect the traits are included in the analysis.

Supplementary Table A2: Results from simulation study with stronger correlations

Parameter	Method	Pruning	Mean	SD	Mean SE	Power
θ_1	MV-IVW	Oracle	0.308	0.182	0.166	47.9
	MV-LIML	Oracle	0.345	0.206	0.184	48.9
	MV-IVW-PCA	-	0.217	0.166	0.153	32.2
	MV-LIML-PCA	-	0.273	0.219	0.167	39.6
θ_2	MV-IVW	Oracle	-0.014	0.182	0.166	7.2
	MV-LIML	Oracle	-0.009	0.209	0.185	6.8
	MV-IVW-PCA	-	-0.027	0.165	0.154	7.1
	MV-LIML-PCA	-	-0.020	0.222	0.167	11.1
θ_3	MV-IVW	Oracle	-0.493	0.181	0.166	82.7
	MV-LIML	Oracle	-0.535	0.204	0.185	84.1
	MV-IVW-PCA	-	-0.387	0.167	0.154	70.7
	MV-LIML-PCA	-	-0.452	0.223	0.168	75.6

Mean estimates, standard deviation (SD) of estimates, mean standard error (mean SE) of estimates, and empirical power of the 95% confidence interval to estimate $\theta_1 = 0.4$, $\theta_2 = 0$, and $\theta_3 = -0.6$. Results for different pruning thresholds are omitted as the correlations between variants were typically all above 0.6.

Supplementary Table A3: Results from simulation study with stronger effects

Parameter	Method	Pruning	Mean	SD	Mean SE	Power
θ_1	MV-IVW	Oracle	0.771	0.214	0.185	96.7
		0.4	0.745	0.265	0.226	87.2
		0.6	0.441	0.341	0.144	81.1
		0.8	−0.565	0.994	0.078	88.5
	MV-LIML	Oracle	0.786	0.230	0.212	94.6
		0.4	0.767	0.312	0.261	82.5
		0.6	0.665	1.787	0.202	81.3
		0.8	0.407	6.488	0.574	87.8
	MV-IVW-PCA	-	0.735	0.209	0.182	95.9
	MV-LIML-PCA	-	0.772	0.257	0.209	92.0
θ_2	MV-IVW	Oracle	0.048	0.209	0.185	9.0
		0.4	0.108	0.265	0.227	11.5
		0.6	0.015	0.295	0.144	28.0
		0.8	−0.353	1.011	0.078	86.7
	MV-LIML	Oracle	0.020	0.220	0.213	5.3
		0.4	0.061	0.306	0.263	8.0
		0.6	−0.355	1.871	0.203	46.0
		0.8	−1.306	6.725	0.575	89.0
	MV-IVW-PCA	-	0.116	0.205	0.182	12.8
	MV-LIML-PCA	-	0.047	0.236	0.209	8.2
θ_3	MV-IVW	Oracle	0.956	0.215	0.185	99.4
		0.4	0.904	0.269	0.228	93.4
		0.6	0.542	0.357	0.144	87.0
		0.8	−0.618	0.987	0.078	87.7
	MV-LIML	Oracle	0.982	0.229	0.213	99.0
		0.4	0.947	0.319	0.263	91.4
		0.6	0.934	1.912	0.202	89.7
		0.8	1.074	6.675	0.575	88.5
	MV-IVW-PCA	-	0.891	0.210	0.183	98.9
	MV-LIML-PCA	-	0.954	0.251	0.210	97.8

Mean estimates, standard deviation (SD) of estimates, mean standard error (mean SE) of estimates, and empirical power of the 95% confidence interval to estimate $\theta_1 = 0.8$, $\theta_2 = 0$, and $\theta_3 = 1.0$. We consider four methods, and various pruning thresholds for the MV-IVW and MV-LIML methods, plus an oracle setting in which only the 15 variants that truly affect the traits are included in the analysis.

Supplementary Table A4: Results from simulation study with alternative correlation matrix ("c-vine")

Parameter	Method	Pruning	Mean	SD	Mean SE	Power
θ_1	MV-IVW	Oracle	0.371	0.137	0.123	83.2
		0.4	0.274	0.114	0.104	73.1
		0.6	−0.135	0.422	0.055	75.6
		0.8	−0.143	0.432	0.054	76.7
	MV-LIML	Oracle	0.386	0.144	0.137	80.9
		0.4	0.351	0.152	0.115	80.9
		0.6	0.131	1.019	0.114	72.8
		0.8	0.131	1.035	0.115	73.5
	MV-IVW-PCA	-	0.334	0.135	0.122	76.2
	MV-LIML-PCA	-	0.368	0.153	0.135	76.6
θ_2	MV-IVW	Oracle	0.002	0.135	0.123	7.3
		0.4	0.002	0.113	0.104	6.9
		0.6	0.006	0.414	0.055	74.4
		0.8	0.005	0.423	0.054	75.5
	MV-LIML	Oracle	0.001	0.143	0.137	5.6
		0.4	0.001	0.154	0.115	13.5
		0.6	0.002	1.056	0.114	71.7
		0.8	0.007	1.104	0.115	71.9
	MV-IVW-PCA	-	0.001	0.134	0.122	6.8
	MV-LIML-PCA	-	0.000	0.153	0.135	7.6
θ_3	MV-IVW	Oracle	−0.558	0.136	0.123	98.8
		0.4	−0.411	0.115	0.104	95.4
		0.6	0.212	0.419	0.055	77.9
		0.8	−0.226	0.427	0.054	79.2
	MV-LIML	Oracle	−0.581	0.141	0.136	98.7
		0.4	−0.529	0.146	0.115	97.6
		0.6	−0.186	0.934	0.113	75.2
		0.8	−0.190	0.970	0.115	75.5
	MV-IVW-PCA	-	−0.502	0.134	0.122	96.6
	MV-LIML-PCA	-	−0.555	0.147	0.135	97.1

Mean estimates, standard deviation (SD) of estimates, mean standard error (mean SE) of estimates, and empirical power of the 95% confidence interval to estimate $\theta_1 = 0.4$, $\theta_2 = 0$, and $\theta_3 = -0.6$. We consider four methods, and various pruning thresholds for the MV-IVW and MV-LIML methods, plus an oracle setting in which only the 15 variants that truly affect the traits are included in the analysis.

Supplementary Table A5: Results from simulation study with alternative correlation matrix ("onion")

Parameter	Method	Pruning	Mean	SD	Mean SE	Power
θ_1	MV-IVW	Oracle	0.374	0.136	0.123	83.6
		0.4	0.276	0.114	0.105	73.6
		0.6	−0.137	0.421	0.055	75.8
		0.8	−0.146	0.434	0.054	77.2
	MV-LIML	Oracle	0.390	0.142	0.137	81.5
		0.4	0.354	0.151	0.115	81.5
		0.6	0.127	1.015	0.114	73.8
		0.8	0.127	1.054	0.115	74.5
	MV-IVW-PCA	-	0.336	0.134	0.122	76.5
	MV-LIML-PCA	-	0.370	0.151	0.135	77.5
θ_2	MV-IVW	Oracle	−0.000	0.137	0.123	7.8
		0.4	−0.000	0.115	0.104	7.5
		0.6	−0.000	0.412	0.055	74.7
		0.8	−0.002	0.423	0.053	75.9
	MV-LIML	Oracle	−0.001	0.145	0.137	6.0
		0.4	−0.001	0.159	0.115	14.6
		0.6	0.004	1.007	0.113	72.0
		0.8	0.005	1.036	0.115	72.8
	MV-IVW-PCA	-	−0.000	0.137	0.122	7.6
	MV-LIML-PCA	-	−0.001	0.158	0.135	8.5
θ_3	MV-IVW	Oracle	−0.556	0.134	0.123	98.7
		0.4	−0.412	0.114	0.104	95.5
		0.6	0.212	0.431	0.055	78.0
		0.8	0.225	0.441	0.053	79.3
	MV-LIML	Oracle	−0.584	0.139	0.137	98.7
		0.4	−0.531	0.146	0.115	97.7
		0.6	−0.189	0.897	0.113	75.2
		0.8	−0.186	0.935	0.115	75.7
	MV-IVW-PCA	-	−0.503	0.133	0.122	96.6
	MV-LIML-PCA	-	−0.556	0.146	0.135	97.2

Mean estimates, standard deviation (SD) of estimates, mean standard error (mean SE) of estimates, and empirical power of the 95% confidence interval to estimate $\theta_1 = 0.4$, $\theta_2 = 0$, and $\theta_3 = -0.6$. We consider four methods, and various pruning thresholds for the MV-IVW and MV-LIML methods, plus an oracle setting in which only the 15 variants that truly affect the traits are included in the analysis.

Supplementary Table A6: Results from simulation study with variant correlations taken from real genetic data.

Parameter	Method	Pruning	Mean	SD	Mean SE	Power
θ_1	MV-IVW	Oracle	0.361	0.135	0.123	81.9
		0.4	0.301	0.134	0.124	67.7
		0.6	0.273	0.120	0.113	67.1
		0.8	0.242	0.113	0.106	63.0
	MV-LIML	Oracle	0.382	0.143	0.137	81.0
		0.4	0.349	0.159	0.136	72.1
		0.6	0.341	0.155	0.123	75.7
		0.8	0.327	0.160	0.115	75.0
	MV-IVW-PCA	-	0.335	0.148	0.137	68.4
	MV-LIML-PCA	-	0.366	0.164	0.151	69.1
θ_2	MV-IVW	Oracle	0.014	0.127	0.116	7.4
		0.4	0.036	0.123	0.115	7.9
		0.6	0.044	0.115	0.108	8.3
		0.8	0.047	0.106	0.100	9.0
	MV-LIML	Oracle	0.005	0.136	0.129	6.1
		0.4	0.019	0.147	0.126	8.9
		0.6	0.027	0.153	0.118	12.7
		0.8	0.031	0.155	0.109	17.1
	MV-IVW-PCA	-	0.022	0.134	0.122	7.3
	MV-LIML-PCA	-	0.008	0.145	0.135	6.2
θ_3	MV-IVW	Oracle	-0.557	0.122	0.112	99.7
		0.4	-0.488	0.118	0.110	99.0
		0.6	-0.454	0.110	0.103	99.0
		0.8	-0.417	0.103	0.097	98.7
	MV-LIML	Oracle	-0.578	0.126	0.124	99.8
		0.4	-0.533	0.132	0.121	99.2
		0.6	-0.523	0.128	0.113	99.3
		0.8	-0.512	0.126	0.106	99.2
	MV-IVW-PCA	-	-0.539	0.126	0.117	99.5
	MV-LIML-PCA	-	-0.565	0.135	0.129	99.5

Mean estimates, standard deviation (SD) of estimates, mean standard error (mean SE) of estimates, and empirical power of the 95% confidence interval to estimate θ_1 = 0.4, θ_2 = 0, and θ_3= -0.6. We consider four methods, and various pruning thresholds for the MV-IVW and MV-LIML methods, plus an oracle setting in which only the 15 variants that truly affect the traits are included in the analysis.

Supplementary Table A7: Results from simulation study with moderately correlated exposures.

Parameter	Method	Pruning	Mean	SD	Mean SE	Power
θ_1	MV-IVW	Oracle	0.354	0.128	0.115	84.6
		0.4	0.305	0.160	0.142	60.3
		0.6	0.210	0.113	0.090	64.7
		0.8	-0.085	0.411	0.049	77.0
	MV-LIML	Oracle	0.391	0.145	0.136	84.0
		0.4	0.361	0.198	0.167	62.3
		0.6	0.398	0.385	0.111	82.0
		0.8	0.113	3.199	0.290	80.8
	MV-IVW-PCA	-	0.299	0.125	0.114	73.2
	MV-LIML-PCA	-	0.381	0.168	0.134	79.8
θ_2	MV-IVW	Oracle	-0.003	0.129	0.115	7.8
		0.4	-0.009	0.162	0.142	7.3
		0.6	0.002	0.107	0.090	9.1
		0.8	0.035	0.409	0.049	75.8
	MV-LIML	Oracle	0.001	0.143	0.136	5.5
		0.4	-0.003	0.205	0.168	7.2
		0.6	0.016	0.368	0.110	28.5
		0.8	0.011	3.264	0.291	80.9
	MV-IVW-PCA	-	-0.009	0.125	0.113	7.5
	MV-LIML-PCA	-	0.000	0.173	0.134	10.3
θ_3	MV-IVW	Oracle	-0.544	0.128	0.115	99.0
		0.4	-0.484	0.161	0.142	89.3
		0.6	-0.310	0.136	0.090	88.2
		0.8	0.221	0.410	0.049	78.5
	MV-LIML	Oracle	-0.587	0.140	0.136	99.0
		0.4	-0.550	0.201	0.168	90.4
		0.6	-0.540	0.355	0.111	94.8
		0.8	-0.071	3.047	0.290	80.8
	MV-IVW-PCA	-	-0.474	0.126	0.114	96.7
	MV-LIML-PCA	-	-0.570	0.165	0.135	97.8

Mean estimates, standard deviation (SD) of estimates, mean standard error (mean SE) of estimates, and empirical power of the 95% confidence interval to estimate $\theta_1 = 0.4$, $\theta_2 = 0$, and $\theta_3 = -0.6$. We consider four methods, and various pruning thresholds for the MV-IVW and MV-LIML methods, plus an oracle setting in which only the 15 variants that truly affect the traits are included in the analysis.

Supplementary Table A8: Results from simulation study with weakly correlated exposures.

Parameter	Method	Pruning	Mean	SD	Mean SE	Power
θ_1	MV-IVW	Oracle	0.344	0.135	0.120	79.3
		0.4	0.286	0.163	0.145	53.7
		0.6	0.186	0.112	0.090	55.1
		0.8	-0.081	0.408	0.049	75.7
	MV-LIML	Oracle	0.381	0.150	0.140	79.3
		0.4	0.340	0.207	0.168	56.7
		0.6	0.327	0.281	0.105	74.3
		0.8	0.070	2.896	0.251	80.8
	MV-IVW-PCA	-	0.279	0.129	0.117	66.0
	MV-LIML-PCA	-	0.356	0.173	0.135	74.1
θ_2	MV-IVW	Oracle	-0.005	0.135	0.120	7.9
		0.4	-0.013	0.165	0.145	7.4
		0.6	-0.002	0.108	0.090	9.3
		0.8	0.039	0.404	0.049	74.7
	MV-LIML	Oracle	-0.001	0.153	0.139	6.2
		0.4	-0.007	0.209	0.168	7.9
		0.6	0.010	0.291	0.105	26.4
		0.8	0.054	3.006	0.252	79.0
	MV-IVW-PCA	-	-0.013	0.129	0.117	7.6
	MV-LIML-PCA	-	-0.004	0.174	0.135	10.9
θ_3	MV-IVW	Oracle	-0.533	0.134	0.120	98.3
		0.4	-0.463	0.166	0.146	85.6
		0.6	-0.286	0.133	0.090	83.8
		0.8	0.216	0.408	0.049	78.0
	MV-LIML	Oracle	-0.575	0.147	0.140	98.3
		0.4	-0.526	0.206	0.169	87.2
		0.6	-0.463	0.325	0.105	92.1
		0.8	-0.056	2.827	0.251	80.7
	MV-IVW-PCA	-	-0.452	0.130	0.117	94.7
	MV-LIML-PCA	-	-0.543	0.168	0.135	96.6

Mean estimates, standard deviation (SD) of estimates, mean standard error (mean SE) of estimates, and empirical power of the 95% confidence interval to estimate $\theta_1 = 0.4$, $\theta_2 = 0$, and $\theta_3 = -0.6$. We consider four methods, and various pruning thresholds for the MV-IVW and MV-LIML methods, plus an oracle setting in which only the 15 variants that truly affect the traits are included in the analysis.

Supplementary Table A9: Results from simulation study with strongly correlated exposures.

Parameter	Method	Pruning	Mean	SD	Mean SE	Power
θ_1	MV-IVW	Oracle	0.363	0.123	0.111	88.5
		0.4	0.321	0.155	0.139	65.8
		0.6	0.233	0.114	0.089	72.8
		0.8	-0.089	0.398	0.049	77.1
	MV-LIML	Oracle	0.399	0.135	0.134	87.0
		0.4	0.380	0.196	0.167	66.4
		0.6	0.463	0.508	0.118	87.5
		0.8	0.163	3.379	0.320	82.0
	MV-IVW-PCA	-	0.317	0.122	0.112	79.4
	MV-LIML-PCA	-	0.402	0.164	0.135	84.2
θ_2	MV-IVW	Oracle	-0.002	0.123	0.111	7.4
		0.4	-0.007	0.158	0.139	7.3
		0.6	0.005	0.108	0.089	9.6
		0.8	0.032	0.397	0.049	76.5
	MV-LIML	Oracle	0.003	0.138	0.134	5.1
		0.4	-0.000	0.199	0.168	6.8
		0.6	0.021	0.441	0.117	29.9
		0.8	-0.013	3.492	0.320	80.8
	MV-IVW-PCA	-	-0.006	0.123	0.112	7.5
	MV-LIML-PCA	-	0.004	0.172	0.135	10.0
θ_3	MV-IVW	Oracle	-0.553	0.124	0.112	99.4
		0.4	-0.501	0.159	0.139	91.3
		0.6	-0.334	0.140	0.090	91.4
		0.8	0.227	0.409	0.049	78.5
	MV-LIML	Oracle	-0.596	0.134	0.134	99.5
		0.4	-0.569	0.200	0.168	91.9
		0.6	-0.609	0.423	0.118	96.5
		0.8	-0.134	3.207	0.319	81.9
	MV-IVW-PCA	-	-0.492	0.124	0.112	97.8
	MV-LIML-PCA	-	-0.591	0.158	0.135	98.6

Mean estimates, standard deviation (SD) of estimates, mean standard error (mean SE) of estimates, and empirical power of the 95% confidence interval to estimate $\theta_1 = 0.4$, $\theta_2 = 0$, and $\theta_3 = -0.6$. We consider four methods, and various pruning thresholds for the MV-IVW and MV-LIML methods, plus an oracle setting in which only the 15 variants that truly affect the traits are included in the analysis.

Supplementary Table A10: Results from the main simulation study with a correlation matrix estimated in an independent sample of 1000 individuals

Parameter	Method	Pruning	Mean	SD	Mean SE	Power
θ_1	MV-IVW	0.4	0.304	0.170	0.148	56.7
		0.6	0.213	0.135	0.085	65.0
	MV-LIML	0.4	0.339	0.199	0.165	58.0
		0.6	0.297	0.191	0.093	77.1
	MV-IVW-PCA	-	0.297	0.132	0.119	69.3
	MV-LIML-PCA	-	0.347	0.155	0.131	74.1
θ_2	MV-IVW	0.4	−0.013	0.170	0.149	8.0
		0.6	−0.012	0.136	0.085	18.7
	MV-LIML	0.4	−0.009	0.198	0.165	7.8
		0.6	−0.002	0.189	0.093	27.8
	MV-IVW-PCA	-	−0.014	0.134	0.119	7.8
	MV-LIML-PCA	-	−0.007	0.160	0.131	9.6
θ_3	MV-IVW	0.4	−0.487	0.169	0.148	86.8
		0.6	−0.355	0.135	0.085	93.2
	MV-LIML	0.4	−0.529	0.193	0.164	87.5
		0.6	−0.459	0.189	0.093	95.7
	MV-IVW-PCA	-	−0.478	0.132	0.119	95.6
	MV-LIML-PCA	-	−0.538	0.154	0.131	96.7

Mean estimates, standard deviation (SD) of estimates, mean standard error (mean SE) of estimates, and empirical power of the 95% confidence interval to estimate $\theta_1 = 0.4$, $\theta_2 = 0$, and $\theta_3 = -0.6$.
